# Geopsychiatry and geography: A response

**DOI:** 10.1177/00207640231195289

**Published:** 2023-10-16

**Authors:** Chris Philo, Felicity Callard, Cheryl McGeachan, Hester Parr

**Affiliations:** School of Geographical and Earth Sciences, University of Glasgow, Scotland, UK

**Keywords:** Mental health geography, geography of mental health, geopsychiatry, interdisciplinarity, geopolitics

## Abstract

**Background:**

This contribution responds to three articles (we refer to all three as ‘editorials’) concerning something called ‘geopsychiatry’.

**Aims:**

To evaluate claims made in these editorials for ‘geopsychiatry’ as a new field of inquiry at the interface between geography and psychiatry.

**Method:**

Close critical reading of two editorials in the *International Journal of Social Psychiatry* – entitled ‘Geographical determinants of mental health’ and ‘Political determinants of mental health’ – and one in the *International Review of Psychiatry* – entitled ‘What is geopsychiatry?’

**Results:**

While this geopsychiatry initiative is to be applauded, disquiet can be expressed about the almost complete neglect of a pre-existing domain of inquiry – ‘mental health geography’ or ‘the geography of mental health’ – that has long been researched by academic geographers and cognate scholars. Key trajectories in this field can be identified and related to the proposed foci for geopsychiatry.

**Conclusions:**

The hope is voiced that future developments in geopsychiatry will proceed in dialogue with the literature and practitioners of mental health geography.

## Geopsychiatry?

This contribution articulates the contributions that the long-standing field of mental health geography can offer to what psychiatric researchers have recently designated as the terrain of ‘geopsychiatry’. An underlying concern is how domains of interdisciplinary inquiry engage with long-standing research expertise in their claimed constituent disciplines, and we consider this question – which involves epistemological and practical concerns – though engaging, as mental health geographers, with claims emerging from psychiatry about this proposed field of geopsychiatry.

We proceed by responding to three editorials that have recently been published, the first of which appeared in the *International Review of Psychiatry* (*IRP*), authored by Castaldelli-Maia and Bhugra (2022), entitled ‘What is geopsychiatry?’. The second two appeared in the present journal, the *International Journal of Social Psychiatry* (*IJSP*), and are respectively entitled ‘Geographical determinants of mental health’ (Bhugra & Ventiglio, 2023a) and ‘Political determinants of mental health’ (Bhugra & Ventigilo, 2023b).^1^ Both of the latter two editorials commence with a reference to ‘GeoPsychiatry’ (with upper-case ‘G’ and ‘P’) – indeed, it is the first word in the text of both pieces – and it is obvious that they sit within the horizon of the 2022 editorial with which they share one author, Bhugra, even though that earlier editorial is unreferenced in either. We should indicate at the outset our enthusiastic embrace of the broad principles underpinning the development of a field that might be called ‘geopsychiatry’. We are in tune with the substance of what the authors argue *should* become central to research and scholarship – and policy and practice applications – that take seriously how geographical considerations shape mental health, mental ill-health, mental healthcare delivery, and more.

Where we have a hesitation, however, is around the ‘geo’ component, since – from our own disciplinary position as academic *geographers* – it is hard for us to shake a sense of ‘reinventing the wheel’, given that there is already a long-standing subfield, with interdisciplinary connections, called ‘mental health geography’ or ‘the geography of mental health’. The authors of the three editorials, and also of related papers, seem unaware of this subfield and of what potentially it could bring to the table of geopsychiatry. In the ‘Geographical determinants of mental health’ editorial (Bhugra & Ventiglio, 2023a), some use is made of Encyclopaedia entries by two academic geographers – with some discussion of Moon (2009) and referencing Elliott (2014) – but these two entries are for ‘health geography’, not specifically ‘mental health geography’.^2^

To be clear, we are not opposed to naming and progressing something called ‘geopsychiatry’ – in fact we like the term – and our hope is to find ways of contributing to this project in future, but we are concerned that due attention be paid to excellent work *already* conducted out of academic geography in *precisely* the ‘space’ for inquiry being identified by the three editorials. The conceptual and empirical resources to be found in geographical literatures offer, we believe, rich ways of envisaging what it might mean to articulate relations between geography and mental health that move beyond what, we feel, are at times rather thin accounts laid out in these three editorials. Geographical research also offers multiple methodological approaches for exploring the complex relationships between these two terms that we believe have much to contribute to geopsychiatry.

### Placing geopsychiatry?

The opening pitch of the *IRP* ‘What is geopsychiatry? editorial (Castaldelli-Maia & Bhugra, 2022) is worth quoting at length because it contains important signposts:

Geopsychiatry is a relatively new and exciting field *in psychiatry*. The discipline studies the interface between *geography and psychiatry*. The main focus in the field is on the impact and effects due to various factors such as climate change, disasters, globalisation, population growth and movement, urban conglomerations, agricultural production, industrialisation, *geopolitics*, socio-economic transformations, and cultural practices in the mental health-mental illness processes. Thus, it is an intersectoral field that involves professionals from varied disciplines such as *geographers*, physicians, anthropologists, sociologists, health professionals (i.e., nurses, social workers, physicians, psychologists, occupational therapists), architects, urban planners, economists, politicians, agronomists among other interested parties. (Castaldelli-Maia & Bhugra, 2022, p. 1; our emphases)

The opening sentence positions geopsychiatry *in psychiatry*, which implies a field of research and application that sits within the orbit of psychiatry, with its own disciplinary and institutional foundations linked to particular research cultures, literatures, philosophies, methods, and expectations about societal impact. The disciplinary background of the editorial’s authors lies in psychiatry and epidemiology, while another individual, Albert Persaud, who has evidently done much to initiate geopsychiatry – to name it, give it shape and direction, and to contribute to associated publications, including ones referenced from the editorial – has a background in clinical psychiatric policy and practice. He is currently the Co-Chair, with one of the editorial authors, João Castaldelli-Maia, of the Working Group on Geopsychiatry (WGG) acting for the World Psychiatry Association.^3^ Unsurprisingly, then, the chief coordinates for this exciting initiative derive from psychiatry and what can be broadly cast as biomedicine, although it must be acknowledged that the literatures consulted in the relevant publications do span quite widely.

The second thing to notice is the reference to *geopolitics*, since the prime focus of the editorial is the realm of geopolitics. Moreover, the website of the WGG^4^ states that ‘[g]eopsychiatry is an exciting developing field and subspeciality in psychiatry’, reinforcing its envisaged home *in* psychiatry, and then explains that ‘[t]he subject focuses on the interface between geopolitical events and psychiatry’. Subsequently, the claim is made that attention to ‘social determinants’ of mental (ill-)health should be supplemented by an awareness of how ‘[g]eopolitical determinants tend to influence social determinants in an increasingly globalised and inter-connected world’. In what is evidently a companion piece to the editorial, the claim is also that ‘[a] geopsychiatry approach frames mental health as being under the ongoing influence of geographically rooted systems of political influence and policy-making’ (Koravangattu et al., 2022, p. 112). This claim sits within a wider recognition of global mental health challenges beyond nation-state boundaries, arising from the horribly complex matrix of what climate change, pandemics, extremism, terrorism, ‘modern slavery’ (also Persaud & Bhugra, 2022), and other phenomena pose for the psychological wellbeing of all, but especially those already most vulnerable due to factors of poverty, ethnicity, gender, disability, and more. Several other papers by Persaud and co-authors very much foreground geopolitics, and one is even entitled ‘Geopolitical factors and mental health’ (Persaud et al., 2018). Repeatedly and rightly emphasised through these geopsychiatric interventions are the mental health implications of human displacement, particularly if forced and accompanied with threats of violence, and the psychological traumas inherent to both pre- and post-migration scenarios.

Such geopolitical themes permeate the *IRP* editorial, but it is interesting how the overall problematic here becomes less ‘geopolitical’ and more broadly ‘geographical’ in its vocabularies. There is the upfront insistence on interfacing between *geography and psychiatry*, followed later with a remark about geopsychiatry attending to ‘the causal chain involving geographic risk factors and incident[s of] mental and substance use disorders’ (Castaldelli & Bhugra, 2022, p. 1). Similarly, the authors mention the relations between urbanisation and mental health, noting that ‘urbanisation may heighten mental illness and psychiatric episodes’ (p. 1), as well as signalling connections between urban environments and ‘stress’. They also propose that rural areas may cause difficulties for mental health, chiefly because of what is commonly the under-servicing of rural areas by mental health treatment and care. As we will elaborate shortly, exploring causal chains between geographical contexts – meaning environments that typically entangle natural and human-made elements – and incidences of mental ill-health (or prevalence of mental well-being) comprises a core strand within geographical work on mental health. In this regard, urban-rural contrasts have often been the bread-and-butter of our inquiries.

While the editorial makes plain that the envisaged new field of geopsychiatry sits primarily within psychiatry, the authors describe it as ‘an intersectional field’ that should be drawing in the expertise of many other ‘professionals’, the first of whom named here are *geographers*. Of course, we are heartened by such an invitation, but there are no signs of ‘us’ yet being enabled to make this contribution, although of course it is ‘early days’ for the field. There do not appear to be any geographers formally associated with the WGG, judging from the membership and corresponding names on the WGG website, and – more problematic for us – there are no geographers (and no publications from academic geographyjournals) cited. The same absence is true of the Koravangattu et al. (2022) chapter, excepting one reference to a piece by Ingram (2005), and also of the ‘Geopolitical factors and mental health’ (Persaud et al., 2018) and ‘Global mental health and climate change: A geopsychiatry perspective’ (Sri et al., 2023) papers, notwithstanding their titles and foci.

### Placing the ‘other’ discipline?

The two *IJSP* editorials are less manifesto-like: indeed, they are essentially very short summary reviews of extant studies that demonstrate, respectively, ‘geographical’ and ‘political determinants’ of mental health. The latter piece essentially rehearses how geographical variations in ‘political systems’ – in ‘government, its ideologies, policies and politics’ – become tied up with ‘various social factors such as poverty, poor housing, unemployment, lack of green spaces, poor public transport and poor access to healthy food or good education’ that all press upon ‘poor mental health’ (Bhugra & Ventiglio, 2023b, p. 1; also Alibudbud, 2023).^5^ It is hence consistent with the geopolitical emphasis that we have already noted. The former piece^6^ offers what we cannot but regard as a highly partial and curiously mixed-up introduction to work in the vein of the famous Faris and Dunham (1939) ‘ecological’ study of schizophrenia in Chicago – tracing the uneven geographical patterning of ‘stressors’ (e.g. low income, unemployment, poor housing, limited service access and more) known to correlate with poor mental health – or echoing a ‘geographical psychology’ (Bhugra & Ventiglio, 2023a, p. 811) that addresses spatial variations in personality types or forms of collective culture as correlated with different mental health states.^7^

The ‘geographical determinants’ editorial also devotes a few words to what the authors understand by the discipline of geography:

Geography [a]s a discipline looks at not only terrain but also environment. Health geography has been described as being akin to medical geography as both subjects look at social models of health and wellness rather than disease and sickness. Health geography also goes beyond traditional biomedical models and looks at the shifting focus to environment as well as geography. (Bhugra & Ventiglio, 2023a, p. 811)

It is hard for us not to feel that our discipline is being somewhat shortchanged by both this description and what then follows. Firstly, geographers past and present tend to regard ‘terrain’, meaning the ‘topography’ of physical landscapes, as a component of the environment, not separate from it, and we would never (at least not without very careful caveating) distinguish – as the authors do later in their editorial – ‘environment’, seemingly understood as the configurations of the ‘natural’ world, from ‘geography’, seemingly taken as variability in human(-made) phenomena such as settlements, agriculture, industry, transport, and more. Secondly, there is a complex genealogy, across several decades, whereby a body of scholarship known as ‘health geography’ has been deliberately carved *apart* from – not positioned as ‘akin to’ – another body of scholarship known as ‘medical geography’ (for an early commentary, see Kearns & Moon, 2002). That ‘carving apart’ has been, at least in part, prompted by an embrace of ‘social models’ that *distance* health geography from the biomedically-inflected model of illness, pathology and clinical intervention typical of medical geography. Thirdly, the editorial authors write something further on about geographers’ interest in ‘place awareness’ that attaches to ‘the physical place and space where people live, work, play and age’ (Bhugra & Ventiglio, 2023a, p. 811), which is nicely put – as too is a remark about how ‘geographical factors . . . affect cultural values as well as socioeconomic and environment factors’ (p. 811) – but we would have to add that *a lot* of demanding, theoretically-informed thinking (across many years of disciplinary self-reflection) lies behind how best to understand ‘space’, ‘place’ and the tangled relationalities of ‘society and space’ and ‘people and place’. To underline, we, as geographers, recognise just how much *more* we need to know before we could begin to encompass psychiatry, its history and theories. And we would have welcomed a little more equivalent circumspection from the editorial authors as they, from their psychiatric background, strive to represent the literatures, debates and accomplishments arising from the discipline of geography.

## Geography?

The issue for us hence becomes why this absence of a more sustained – and, dare we say, more informed – engagement with geography and geographers from an initiative so transparently badged as about ‘geography and psychiatry’ and stating that ‘geographers’ should be part of the picture. This is not the occasion to provide a detailed resumé of past or ongoing geographical work on mental health, but it is worth underscoring that a subfield of academic geography variously named ‘geography and mental health’ (Smith, 1977), ‘the geography of mental health’ (Smith, 1978) or ‘mental health geograph/ies’ (Lowe et al., 2014; c.f. Philo, 2014) *has* arisen and become well-known. Book-length monographs or collections have delimited and developed this subfield (e.g., Curtis, 2010; Dear & Taylor, 1982; Dear & Wolch 1987; Smith & Giggs, 1988; Parr, 2008; Philo, 2004) while review articles can be cited (McGeachan & Philo, 2017; Philo, 1986, Part 3, 1997, 2005; Wolch & Philo, 2000). Philo (2005) explicitly introduced the field to psychiatrists with his review published in *Current Opinion in Psychiatry*, while, intriguingly, there have been occasional accounts given by individuals outwith academic geography (e. g., Holley, 1998; P. A. Jones, 2007) directly addressing journals specialising in psychiatry and mental health. There is hence a sizeable and easily accessible literature base that, a priori, we might have expected to be at least name-checked in the three editorials and other articles mentioned above.

There have been two principal trajectories within this field. The first – indebted to the Faris and Dunham (1939) study that is referenced by Bhugra and Ventriglio (2023a, p. 812) – has tackled the geographical incidence of mental unwellness, typically mapped using home addresses of individuals clinically diagnosed with ‘mental illness’. The iconic geographical study here is Giggs (1973) on the inner-city ‘production’ of schizophrenia in Nottingham, UK, but Giggs – as Faris and Dunham before him – was cognisant of the complication that people with serious mental health problems might not become unwell in situ, but rather may do so elsewhere and then ‘drift’ into inner-city areas where sources of support, formal and informal, are more readily available. The point here is less the specifics of such studies, though, more that they index a substantial body of research – by geographers and cognate scholars – that does indeed explore the ‘casual chain’ from environmental surroundings into the psychological states – healthy and damaged, happy and traumatic – of many different human groupings in many different times and places.^7^ It is worth adding that very recent endeavours in this vein have attended to the geographical relations and connections intimately folded into the likes of austerity (e.g. Lowe & DeVerteuil, 2020), pandemic (e.g. Andrews et al., 2021), climate change (e.g. Boyd et al., 2023) and forced migrations. Countless inquiries, moreover, including many by geographers who might not even identify with the subfield in question, have addressed precisely the kinds of geographical contexts – including fraught geopolitical or geosocial contexts of displacement, insecurity and trauma (e.g. Ehrkamp et al., 2022; Loyd et al., 2018; Proudfoot, 2019) – that the editorial (and Persaud and co-workers) identify as the foci for geopsychiatry.

The second principal trajectory within geographical work on mental health arguably stirs something different again into the equation. This trajectory explores the multiple geographies bound into the sites, settings and structures (buildings and interiors) that have figured over the centuries in the cure, care, shelter and sometimes restraint of people with mental health problems. In particular, it has considered the transition in many Global-Northern regions from sizeable ‘lunatic asylums’ or mental hospitals, often sited in rural locations, to a kaleidoscope of deinstitutionalised services peppering ‘everyday’ communities and neighbourhoods, notably as dispersed across urban areas. A shorthand to encapsulate this transition speaks of a shift from ‘asylum geographies’ to ‘post-asylum geographies’ (Philo, 2000), recognising the press of globe-sweeping forces and ideologies – financial crises, neoliberalism, ‘big pharma’, racism, sexism – in shaping the exact trajectories of who has been affected, how much, when and where. The optic has often been ‘landscapes of despair’ (Dear & Wolch, 1987), alert to the stigmatised life-worlds under study and the relative powerlessness of the peoples concerned to contribute meaningfully to their *own* ‘place-making’ (e.g. Kiely, 2021; McGeachan & Philo, 2023).

The two trajectories identified here have tended to lay emphasis on what might be termed the ‘external’ or ‘structural’ conditions – always constituted in particular ways within given geographical contexts and themselves diffusing unevenly across space – that inextricably shape both the occurrence of mental (ill-)health and the composition of its societal responses (in or out of institutional settings). What geographers have also increasingly offered is a window into the ‘internal’ or ‘agentic’ worlds of people who acquire mental health problems, seeking to grasp how the immediacies of their environmental surroundings – their places, to put it simply – may be harming their psychological well-being or, more optimistically, serving to protect or even strengthen that well-being. A commitment has arisen to engaging with the lived experience of mental (ill-)health, sometimes exemplified in the most demanding of participative work within the everyday places of life, survival, activity and encounter for those enduring troubled mental health. Such work has taken geographers into ‘back wards’, day centres, drop-ins, cafés, parks, streets and many more places, sometimes using inventive art-based methods for approaching and representing the place-person dynamics in play. Some notable PhD theses in this respect include Kiely (2022), Laws (2012), Liggins (2016), Martin, (2021), and McDougall (2021). Parr’s more ‘hopeful’ account of grassroots initiatives in deinstutionalised mental healthcare can also be mentioned here, where ethnographic involvement discloses how gardening, arts and virtual initiatives may aid in the individual ‘recovery’ of some mental health patients, as well as contributing to a wider social ‘rescripting’ of the figure of the mental health patient that promises less-stigmatised futures (Parr, 2008; also Evans & Wilton, 2019).

There is, therefore, *already* this vibrant field of academic inquiry comprising geographical work on mental health, itself informed by a diversity of conceptual approaches – borrowing from a diversity of philosophies and social theories – and deploying all manner of methodologies from the most quantitative-survey-based to the most qualitative and experiential.^8^ The conceptual, methodological and empirical frameworks developed over many decades by geographers, alongside anthropologists, sociologists, and other interpretive social scientists, have much to offer – not least when informed by ongoing efforts to outline how interdisciplinary research might do justice to diverse epistemic backgrounds (e.g. Callard & Fitzgerald, 2015).^9^ We must be honest and acknowledge that an element of our own response here is hence ‘flag-waving’: we *are* here, and we feel that this new proposal for a geopsychiatry operating at the interface between geography an psychiatry probably should have known that we are here, or at least now look with curiosity at what we have already achieved and may have to offer. But we are not disciplinary imperialists: we are not suggesting that academic geography should comprise the meta-disciplinary home for geopsychiatry, rather than psychiatry, and we are not in the business of jealously protecting – nor acquisitively expanding – our intellectual ‘turf’. Rather, we submit this response in the spirit of reaching out to what the PhD supervisor of one of us once felicitously termed ‘geographers in other disciplines’, meaning like-minded scholars who themselves have come to the conclusion that ‘geography matters’ (Massey & Allen, 1984) to whatever issues and problems preoccupy them. Clearly the latter is true of the authors of the three geopsychiatry editorials, and we hope that mental health geographers will be able to dialogue with them in this important new venture.

## Coda

Lynne Jones’s memoir of practising ‘humanitarian psychiatry’ in war and disaster zones (L. Jones, 2018) talks of taking the best principles of ‘asylum’ – sanctuary, refuge, respite, care, kindness and even cure – ‘outside the asylum’ into the usually ramshackle and unprepossessing spaces (lean-tos, camps, roadsides) where she and dedicated colleagues simply did their best. It is a memoir full of rich geographical descriptions, one abiding message being that ‘geography matters’ here: it makes a huge difference in terms of both the (often geopolitically-forced) traumatic situations involved and the halting possibilities for successfully enacting ‘front-line’ psychiatry. That this is the case is perhaps unsurprising because Jones took an undergraduate geography degree at the University of Oxford, the reasons for which she nicely recounts early in her memoir, touchingly recalling a school Geography teacher who ‘taught [her] to take pleasure in understanding how the landscape, both natural and human, was formed’ (p. 2).^10^ What she also recalls is the disdain that she met during her first interview for a psychiatric post: ‘I see you started off in geography, Dr Jones – what took you into medicine? A balding man with heavy brows made studying geography sound akin to working in a strip club’ (p. 1). Jones hence faced the prejudice of a scientifically-trained medical *man* unable to see why a geographical sensibility – which she subsequently, knowingly, conjoined with readings in anthropology and a move into medical training^11^ – might have anything to do with psychiatry. She clearly rebelled against that prejudice, retaining an acute readiness to be, in effect, a geographer within psychiatry: we might call her an exponent, a most compelling one, of geopsychiatry in action. Her example is a highly appropriate coda to the claims that we have been advancing above in this response to the geopsychiatry editorial.

## Notes

1. A third *IJSP* editorial (Bhruga & Ventriglio, 2023c) discusses ‘Commercial determinants of mental health’, chiefly addressing how the production and consumption – specifically advertising – components of multi-national corporations (MNCs) potentially impacts, in a negative fashion, many people (mainly consumers, but there is also some welcome acknowledgement of how workers [employees] might be affected). From our perspective, there are multiple economic geographies of MNCs – and more broadly of capitalism in its various varieties and phases – indelibly bound into these ‘commercial determinants’ of poor mental health.

2. Alongside Persuad and Castaldelli-Maia, Bhugra is Committee Member of the WGG, while Ventriglio, co-author of the two *IJSP* editorials, is a Group Member of the WGG. Information in links from https://www.wpanet.org/wg-on-geopsychiatry.

3. See front page of https://www.wpanet.org/wg-on-geopsychiatry.

4. ‘Health geography’ is conventionally identified as one of the subdisciplines of academic geography, paralleling, even supplanting, ‘medical geography’, while ‘mental health geography’ (or some equivalent name) is normally taken as a subfield of ‘health geography’ (sometimes connecting across to other subdisciplines such as ‘social geography’, ‘cultural geography’ and ‘historical geography’, as well as to fields outwith the discipline such as psychiatry: and there is an intriguing minor history to be written of geographers and psychiatrists working together).

5. This editorial commences with: ‘GeoPsychiatry as an emerging special[ity] within psychiatry which aims to bring together sociology, anthropology, political sciences, psychiatry, medicine and other stake-holders in order to understand the impact of *geo-political* determinants on health including mental health’ (Bhugra & Ventiglio, 2023b, p. 1: our emphasis). ‘Geography’ as a stake-holder has gone missing here, while the bottom-line focus appears once again to be *geo*political.

6. This editorial commences with: ‘GeoPsychiatry is the branch of psychiatry which aims to study determinants which influence social determinants which in turn affect physical and mental health of individuals as well as populations. Although increasing attempts are being made, geographical determinants are not well studied’ (Bhugra & Ventiglio, 2023a, p. 811). The root claim is that there are ‘geographical determinants’ – geographically variable influences, causes or ‘drivers’ – shaping ‘social determinants’ of mental health. We might suggest the need for a more sinuous, conceptually-informed vocabulary here that would speak of geographical and social dimensions mutually influencing one another – what we sometimes term a ‘socio-spatial dialectic’ – and having diverse impacts upon the constitution of mental (ill-)health. We would also suggest that there *is* already an extant body of work exploring these ‘geographical determinants’, if not always expressing itself as such, to the extent that it is arguably a mis-step to claim that such determinants are ‘not well studied’.

7. We might add that geographers have engaged in various ways with ‘psychology’ – a discipline that must not be conflated with psychiatry, notwithstanding its own clinical wing – considering spatial variability in personality types and all manner of ways in which environmental contexts, widely defined, may shape human-psychological processes, including senses of well- or ill-being. Approaches variously called ‘behavioural’, ‘cognitive’ and ‘perceptual geographies’ have played a role here, sometimes drawing explicitly from the subfield of psychology known as ‘environmental psychology’. A further angle again has been the development of ‘psychoanalytic’ and ‘psychotherapeutic geographies’. We would not expect the authors of the editorials under review to know such details per se, but they might have anticipated that a mature academic discipline such as geography would, at some moments in its historical and theoretical development, have looked across in detail at the various ‘psy’-disciplines.

8. While hesitant about picking out particular scholars, we do wish to flag the contributions of Steven Cummings, a geographer with epidemiological training, who has led or contributed to quantitative – but often conceptually eclectic – inquiries into all manner of geographically-distributed social phenomena (housing types, food availability, ‘green space’, urban regeneration, major sports event and more) and their complex health effects for physical and especially psychological well-being. See https://www.lshtm.ac.uk/aboutus/people/cummins.steven#publications.

9. We fully acknowledge that the subdisciplinary field still has much to do in diversifying from its Global-Northern contexts – in terms of both where substantive inquiries are focussed and who is conducting such inquiries from which institutional bases – and in this respect geopsychiatry (as envisaged in this editorial and related initiatives) may have much to teach and offer us.

10. ‘Tramping the chalk hills and valleys of the South Downs, mapping villages, tracing their development and interviewing residents about their lives . . .’ (L. Jones, 2018, p. 2).

11. We should admit that Jones did not stay with her geography programme for long: ‘Studying timber exports from Norway in my first year was boring’, and she switched to a new interdisciplinary degree in Human Sciences where she found ways to interface geography, anthropology and other disciplinary perspectives. She specifically mentions being inspired by ‘the Villas-Bôas brothers, two geographers and anthropologists, who recognised that remote tribes in Brazil needed shielding from the encroachments of Western civilization and so became advocates and activities to protect them. They also set up clinics to address the ravages of Western illnesses such as measles. I suddenly realised that if I trained in medicine, I could combine it with anthropology and geography’ (L. Jones, 2018, p. 3).
